# Establishment of a staging system for visceral sarcoma

**DOI:** 10.1002/cam4.6791

**Published:** 2023-12-16

**Authors:** Bingnan Wang, Lun Xu, Meng Fang, Biqiang Zheng, Wangjun Yan

**Affiliations:** ^1^ Department of Musculoskeletal Oncology Fudan University Shanghai Cancer Center Shanghai China; ^2^ Department of Oncology, Shanghai Medical College Fudan University Shanghai China

**Keywords:** prognosis, soft tissue sarcoma, stage, visceral sarcoma

## Abstract

**Background:**

Visceral sarcoma is a rare malignancy with a poor prognosis. However, there is no recommended prognostic staging system for the malignant disease.

**Method:**

We analyzed the data of patients diagnosed with primary soft tissue sarcoma (STS) of the abdomen and thoracic visceral organs between 2006 and 2017 at our hospital. Prognostic factors (size, tumor grade, and lymph node metastasis) were analyzed in our cohort (*n* = 203) and the SEER validation cohort (*n* = 5826).

**Results:**

Tumor size, grade, and lymph node metastasis were important prognostic factors for visceral sarcoma in both our and the SEER cohorts. Based on these prognostic factors, we established a new staging system for visceral sarcoma, by which patients could be stratified into clinically meaningful and non‐overlapping stages in both our cohort and the SEER validation series. Moreover, the area under the curve (AUC) value of the staging system for 5‐year survival was 0.84 (95% CI: 0.78–0.89) in our series and 0.80 (95% CI: 0.79–0.81) in SEER series, respectively. In addition, compared with the widely used FIGO staging system for female genital sarcoma, the visceral sarcoma staging system could more effectively and reliably stratify patients into four different prognostic groups.

**Conclusions:**

The visceral sarcoma staging system is applicable for STS of the abdomen and thoracic visceral organs and is better than the current FIGO staging system for female genital sarcoma and should be incorporated into the AJCC Cancer Staging Manual.

## Graphical abstract

We established a new staging system for visceral sarcoma, by which patients could be stratified into clinically meaningful and non‐overlapping stages in both our cohort and the SEER validation series. The prognosis of visceral sarcoma can be stratified by the staging system regardless of location within the body. The visceral sarcoma staging system is better than the current FIGO staging system for staging female genital sarcoma.

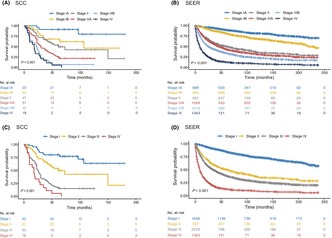

## 1 INTRODUCTION

Soft tissue sarcomas (STS) are rare malignancies, accounting for approximately 1% of adult malignant tumors.^1^ STS can occur in various locations of the body, with the extremities (40%), trunk (10%), viscera (22%), and retroperitoneum (16%) being the most common primary sites.^2^ Due to the rarity and distribution of different visceral organs, sarcomas from the viscera (except gastrointestinal stromal tumors) are difficult to study as a group compared with those from other sites. Visceral sarcomas show poor prognosis^3^, ^4^, ^5^ and have significantly worse outcomes than extremity/trunk sarcomas.^4^, ^6^, ^7^ One of the important reasons is that there is no recommended prognostic staging system for visceral sarcoma from the AJCC Cancer Staging Manual,^8^ which leads to difficulty in predicting patient outcomes and planning for adjuvant therapy.

The clinical staging of cancer is fundamentally important when discussing patient outcomes.^9^ For STS, the TNM stage classification is dependent on the site of the primary tumor according to the NCCN Clinical Practice Guidelines,^10^ and four tumor locations are described: (1) head and neck, (2) trunk and extremity, (3) abdominal and thoracic visceral organs, and (4) retroperitoneum.^8^ However, there is no anatomic prognostic stage for STS of the viscera or head and neck, although the TNM classification of tumor grade has been presented.^10^ For visceral sarcoma, the definition of T categories is based on the scope of adjacent organ invasion,^8^ which shows limited clinical value and ignores the importance of tumor size and grade on prognosis.^5^ Tumor size and grade are important prognostic indicators in patients with visceral sarcoma.^3^, ^4^, ^7^ Even for uterine sarcomas, the current International Federation of Gynecology and Obstetrics (FIGO) staging system cannot effectively and reliably stratify patients into four different prognostic groups; it neglects the importance of tumor grade and size for patient survival.^11^, ^12^, ^13^, ^14^, ^15^ Therefore, the establishment of a staging system for STS of the abdomen and thoracic visceral organs is urgently needed.

In this study, we found that tumor size, histologic grade, and lymph node involvement could significantly influence overall survival for visceral sarcoma in both our (*n* = 203) and the Surveillance, Epidemiology, and End Results (SEER) validation cohort (a large, multi‐institutional database, *n* = 5826). Based on these prognostic factors, we established a new staging system for visceral sarcoma. We subsequently analyzed the performance of the staging system in two separate series. To test the effectiveness of the staging system, we compared it with the widely used FIGO staging system for female genital sarcomas to determine which was better.

## 2 MATERIALS AND METHODS

### 2.1 Patient cohort

Patients with primary sarcomas of the abdomen and thoracic visceral organs and those undergoing surgery between June 2006 and December 2017 were included. The last follow‐up date was June 2022. Patients were excluded based on the following criteria: (1) the sarcoma was an organ metastasis, not primary; (2) the tumor was diagnosed as a gastrointestinal stromal tumor or carcinosarcoma; or (3) the tumor originated from the superficial organs (breast, testis, or penis). After screening, a cohort of 203 patients were included. Tumors were divided into three grades according to the Federation Nationale des Centres de Lutte Contre la Cancer (FNCLCC) criteria.^16^ All patients provided written informed consent, and the study was approved by the Clinical Research Ethics Committee of Fudan University Shanghai Cancer Center (Number: 050432‐4‐1805C).

In addition, we selected patients from the SEER database, which contains data on cancer occurrence in 17 areas of the United States. After screening, 5826 eligible cases registered between 2000 and 2017 were included. Detailed information on the selection process is presented in the Supplementary Material and Figure S1.

### 2.2 Statistical analysis

Survival time was calculated from the date of surgery to the date of last follow‐up or date of death. Overall survival was analyzed using Kaplan–Meier curves. The log‐rank test was used to detect differences in survival between the staging groups and evaluate prognostic factors, including tumor grade, tumor size, and lymph node metastasis. Multivariate analysis was performed using Cox proportional hazards regression with hazard ratios (HRs), and 95% confidence intervals (CIs) were calculated. The area under the receiver operating characteristic (ROC) curve (AUC) was used to evaluate the clinical performance of the visceral sarcoma staging classification in predicting survival at various time points. All statistical analyses were conducted using R‐4.2.0 software. Statistical significance was defined as a two‐sided *p* < 0.05.

## 3 RESULTS

### 3.1 Patient characteristics

A total of 203 patients at the Shanghai Cancer Center (SCC) were included in the study. The clinical characteristics of the patients are provided in Table 1. Visceral sarcomas were most common in the female genital system (*n* = 135), followed by the digestive (*n* = 24) and urinary systems (*n* = 23). The most common malignant pathological types were leiomyosarcomas (*n* = 66), adenosarcomas (*n* = 29), and undifferentiated pleomorphic sarcoma (UPS, *n* = 28). Sarcomas from uncommon sites with histopathology were shown in Supplemental Table S1. The SEER database (Table 1) was used as the validation cohort. Detailed information on the selection process is presented in the Supplementary Material and Figure S1. The 5‐year survival rate for visceral sarcoma from SCC and SEER was 38.3% and 46.7% respectively.

**TABLE 1:** Clinical characteristics of study population.

Variable	SCC	SEER
	No. of Cases (%)	No. of Cases (%)
Age (years)		
≤ 50	85 (41.9%)	1966 (33.7%)
>50	118 (58.1%)	3860 (66.3%)
Sex		
Male	40 (19.7%)	917 (15.7%)
Female	163 (80.3%)	4909 (84.3%)
Tumor location		
Female genital system	135 (66.5%)	4133 (70.9%)
Digestive system	24 (11.8%)	763 (13.1%)
Urinary system	23 (11.3%)	448 (7.7%)
Respiratory system	16 (7.9%)	393 (6.8%)
Others	5 (2.5%)	89 (1.5%)
Histological type		
Leiomyosarcoma	66 (32.5%)	2412 (41.4%)
Adenosarcoma	29 (14.3%)	418 (7.2%)
UPS	28 (13.8%)	140 (2.4%)
ESS	18 (8.9%)	1428(24.5%)
Rhabdomyosarcoma	14 (6.9%)	116 (2.0%)
Liposarcoma	10 (4.9%)	123 (2.1%)
Other	38 (18.7%)	1189 (20.4%)
Tumor grade		
Low grade	47 (23.2%)	1872 (32.1%)
High grade	156 (76.8%)	3954 (67.9%)
Tumor size (cm)		
≤5 cm	75 (36.9%)	1473 (25.3%)
>5–10 cm	72 (35.5%)	2249 (38.6%)
>10–15 cm	38 (18.7%)	1291 (22.2%)
>15 cm	18 (8.9%)	813 (13.9%)
LNM	11 (5.4%)	593 (10.2%)
Distant metastasis	18 (8.9%)	1343 (23.1%)
Visceral sarcoma staging system		
Stage I A	23 (11.3%)	698 (12.0%)
Stage I B	22 (10.8%)	950 (16.3%)
Stage II	47 (23.2%)	557 (9.5%)
Stage III A	51 (25.1%)	1059 (18.2%)
Stage III B	42 (20.7%)	1219 (20.9%)
Stage IV	18 (8.9%)	1343 (23.1%)

Abbreviations: ESS, endometrial stromal sarcoma; LNM, lymph node metastasis; SCC, Shanghai Cancer Center; SEER, Surveillance, Epidemiology, and End Results; UPS, undifferentiated pleomorphic sarcoma.

### 3.2 Tumor size, grade, and lymph node metastasis are important prognostic factors for visceral sarcoma

**FIGURE 1:**
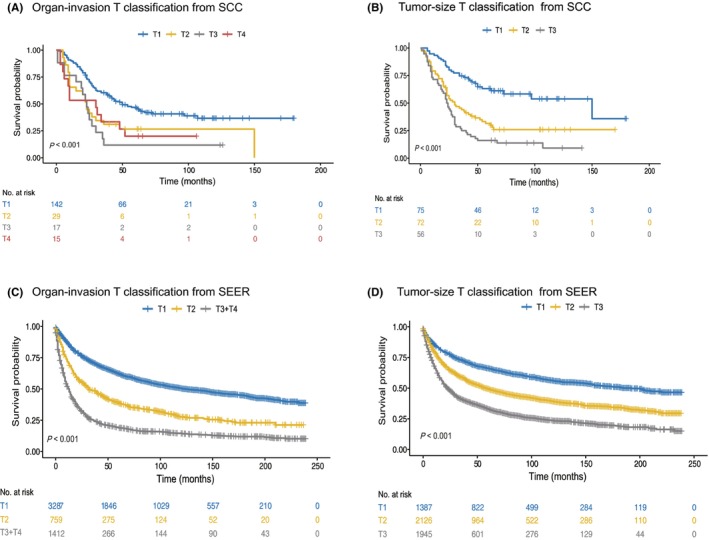
Comparison of the organ‐invasion T classification with the tumor‐size T classification. The patients with visceral sarcoma from the SCC and SEER databases were, respectively, classified by the organ‐invasion T (A, C). The same patients from the SCC and SEER databases were, respectively, re‐classified by the tumor‐size T (B, D).

According to the NCCN TNM Staging of visceral sarcoma, no prognostic stage groupings are provided, but the proposed tumor (T) classifications based on organ confinement and multifocality (organ‐invasion T) are presented.^10^ However, there is no evidence of the clinical utility of T classification for visceral sarcoma.^7^ Instead, studies have shown that tumor size (tumor‐size T) is an important clinicopathological prognostic factor for visceral sarcoma.^3^, ^4^, ^5^ To determine which T classification is a better‐fitting model for visceral sarcoma, we compared the performance of organ‐invasion T with tumor‐size T in stratifying the risk of death. As the multifocality scheme cannot be applied to the SEER cohort because no related data on multifocality exists, STS involvement of adjacent organs was classified in the T3 + T4 category in the SEER database. Classified by organ‐invasion T, we found that T1 disease accounted for a large proportion in both the SCC (Figure 1A; T1, 70.0%) and SEER series (Figure 1C; T1, 60.2%). However, the tumor‐size T classification could stratify visceral sarcoma more evenly than the organ‐invasion T classification not only in the SCC but also in the SEER cohort (Figure 1B,D). The prognosis of tumor‐size T1 was better than that of organ‐invasion T1 (5‐year survival: 61.2% vs. 46%, respectively, in SCC; and 66.1% vs. 62.3%, respectively, in SEER) (Figure 1B,D). In the organ‐invasion T classification, we also observed that the prognosis was worse when the T category was higher (Figure 1C). To explore the possible reasons for this, we analyzed the parameters of tumor size and grade between the T3 + T4 and T1 + T2 groups. Compared with the T1 + T2 group, we found that high T (T3 + T4) was associated with large tumor size and high grade in both cohorts (Figure S2).

**FIGURE 2:**
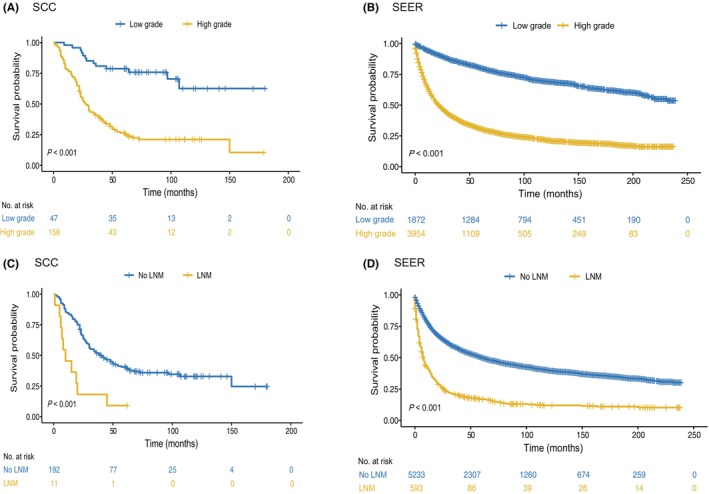
Tumor grade and lymph node metastasis are important prognostic factors for visceral sarcoma. The patients with visceral sarcoma were stratified by tumor grade (A, B) and lymph node metastasis (LNM) (C, D), from the SCC (A, C) and SEER cohorts (B, D).

Previous studies have identified histologic grade as one of the most significant independent adverse predictors for patients with primary sarcoma of the lung,^4^ genitourinary organs,^3^ and uterus.^17^ This was also confirmed by the analysis of our data (Figure 2A), with 5‐year survival rate of 26.5% and 78.7% for high‐ and low‐grade patients, respectively. In the SEER database, tumor grades were classified into four categories (G1, G2, G3, and G4), which was not based on the Federation Nationale des Centres de Lutte Contre la Cancer (FNCLCC) criteria.^16^ We first compared the prognoses of the four categories G. The results showed that G1 patients had similar survival rates to G2 patients, and G3 and G4 patients showed a closed prognosis (Figure S3). Therefore, we attributed G1 and G2 as low grade and G3 and G4 as high grade, which is also in accordance with Spraker and Koshy's study.^4^, ^18^ In line with our results, patients with high‐grade STS showed significantly worse prognosis than those with low‐grade STS in the SEER cohort (Figure 2B, The 5‐year survival rate, 30.9% vs. 80.1%, respectively). In addition, we found that lymph node involvement was an important prognostic factor in both the SCC and SEER databases (Figure 2C,D).

### 3.3 Establishment of a staging system for STS of the abdomen and thoracic visceral organs

Tumor size, grade, and lymph node metastasis are all important prognostic factors for visceral sarcoma and can markedly influence survival rates (Figures 1B,D, and 2). It is well known that the staging of STS from the trunk/extremity or retroperitoneum is based on parameters such as tumor size, grade, and lymph node metastasis,^8^ so we hypothesized that visceral sarcoma may be staged in a manner similar to STS from the trunk/extremity or retroperitoneum. Notably, there is a slight difference between the trunk/extremity and retroperitoneum staging systems; lymph node metastasis is classified as stage IIIB in retroperitoneal STS and as stage IV in trunk/extremity STS.^10^ Next, we investigated whether lymph node involvement belongs to stage IIIB or IV for visceral sarcomas. The proportion of lymph node metastases was 5.4% in SCC and 10.2% in SEER (Figure S4A). In our data, a total of 11 patients had lymph node metastasis (Table 1). Among them, there was only one patient with both lymph node and distant metastasis. In the SEER database, 593 patients were positive for lymph node metastasis. Among them, 318 patients (53.6%) had distant metastasis (Figure S4B) and were classified as stage IV according to current staging in the STS of the trunk/extremity or retroperitoneum; these patients showed the worst outcome in all stages (Figure S4D). The survival curve of lymph node metastasis (*n* = 275) was closer to stage IIIB than to stage IV in the SEER cohort (the 5‐year survival rate for patients with IIIB, lymph node metastasis and IV was 35.6%, 27.5% and 12.7% respectively), although this result was not observed in our cohort due to the limited number of cases (Figure S4C). Therefore, it is more reasonable to classify lymph node‐positive tumors as IIIB than as stage IV.

**FIGURE 3:**
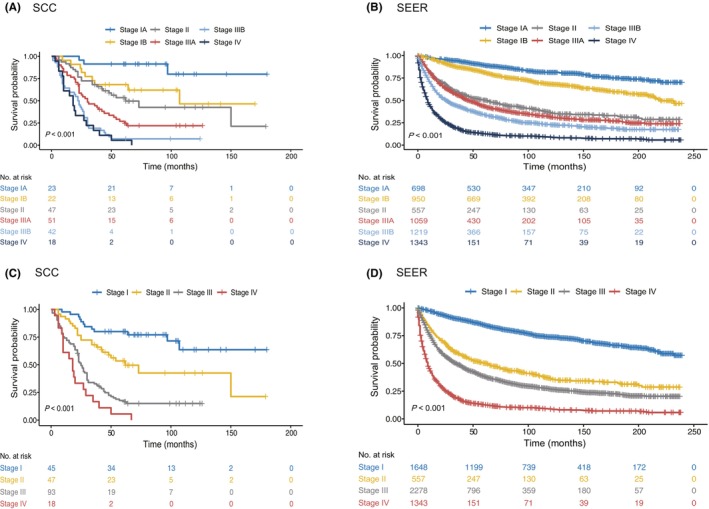
The classification of visceral sarcoma by the new visceral sarcoma staging system. The patients with visceral sarcoma were classified as stage IA, IB, II, IIIA, IIIB, or IV from the SCC database (A) and the SEER validating database (B). Patients with visceral sarcoma were classified as stage I, II, III, or IV from the SCC database (C) and the SEER validating database (D).

With the establishment of a new staging system for visceral sarcoma (Table S2), the patients were classified into different and meaningful TNM stages (IA, IB, II, IIIA, IIIB, IV, Figure 3A,B). The 5‐year survival rate was 91.3%, 68.2%, 53.4%, 26.9%, 7.1%, and 5.6% for stage IA, IB, II, IIIA, IIIB, and IV, respectively, in the SCC cohort; for SEER cohort, the 5‐year survival rate was 89.2%, 81.0%, 50.3%, 43.5%, 33.9%, and 12.7% for stage IA, IB, II, IIIA, IIIB, and IV, respectively, in the SEER database. Furthermore, the visceral sarcoma staging system stratified patients evenly across stages not only in our cohort, but also in the SEER cohort (Table 1). In addition, patients showed significantly different and non‐overlapping prognoses in stages I, II, III, and IV in the two series (Figure 3C,D).

**FIGURE 4:**
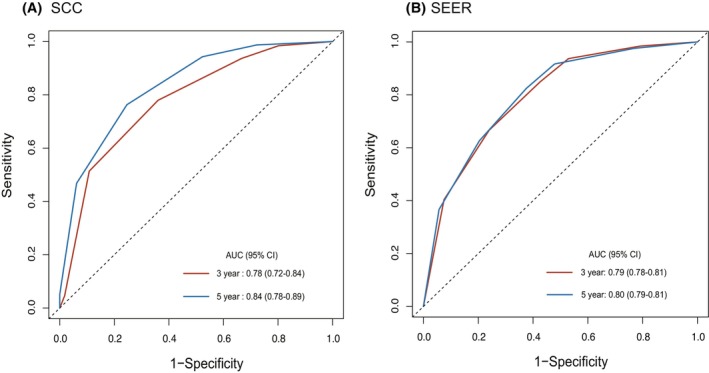
ROC curves based on the visceral sarcoma staging system from SCC cohort (A) and SEER validating cohort (B). ROC: receiver operating characteristic; AUC: area under the curve.

Multivariate analysis showed that age and stage were independent poor prognostic factors in both cohorts (Table 2). Age > 50 years was associated with poor prognosis. Female patients showed a lower HR than males in SEER (HR = 0.84, *p* = 0.004), but this was not observed in our patients (HR = 1.2, *p* = 0.25). There were no consistent correlations between the tumor site, histological type, and HR in either database. Importantly, apart from stage IV in our cohort, multivariate analysis showed that the visceral sarcoma staging system was strongly associated with a stepwise incremental increase in the hazard of death as the stage increased in both cohorts (Table 2). In addition, time‐dependent ROC curves showed that the visceral sarcoma staging system has excellent 3‐ and 5‐year predictive abilities for survival outcomes (Figure 4). The AUCs for the 3‐ and 5‐year survival were 0.78 (95% CI: 0.72–0.84) and 0.84 (95% CI: 0.78–0.89) in SCC series, and 0.79 (95% CI: 0.78–0.81) and 0.80 (95% CI: 0.79–0.81) in SEER series, respectively, which indicates good discriminative ability of the TNM stage for visceral sarcoma.

**TABLE 2:** Multivariate Cox regression analysis of overall survival in visceral sarcoma.

Variable	SCC		SEER	
	HR (95% CI)	*p* value	HR (95% CI)	*p* value
Age				
≤50	Reference		Reference	
>50	1.65 (1.24–2.02)	0.02	1.92(1.77–2.09)	<0.001
Sex				
Male	Reference		Reference	
Female	1.20 (0.79–2.47)	0.25	0.84(0.75–0.95)	0.004
Site				
Female genital system	Reference			
Urinary system	1.10 (0.67–2.87)	0.77	0.97 (0.83–1.12)	0.62
Respiratory system	1.03 (0.32–1.67)	0.43	1.21 (1.04–1.41)	0.01
Digestive system	1.12 (0.57–2.21)	0.73	0.90 (0.80–1.02)	0.09
Others	3.75 (1.35–10.41)	0.06	0.94 (0.71–1.23)	0.48
Histological type				
Leiomyosarcoma	Reference		Reference	
Rhabdomyosarcoma	0.85 (0.60–1.21)	0.12	1.41 (1.13–1.74)	0.002
UPS	1.69 (1.01–2.84)	0.04	1.14 (0.93–1.40)	0.26
Adenosarcoma	0.95 (0.52–1.72)	0.87	0.87 (0.75–1.03)	0.11
Liposarcoma	1.25 (0.59–2.66)	0.56	1.00 (0.78–1.28)	0.91
ESS	0.64 (0.29–1.39)	0.08	0.74 (0.67–0.83)	<0.001
Other	0.97 (0.55–1.72)	0.93	1.47 (1.34–1.62)	<0.001
Stage				
IA	Reference		Reference	
IB	3.48 (1.13–10.04)	0.06	1.70 (1.38–2.10)	<0.001
II	5.18 (1.81–15.89)	0.01	3.38 (2.74–4.17)	<0.001
III A	11.43(3.25–32.77)	<0.001	4.07 (3.35–4.94)	<0.001
III B	21.34 (8.60–56.20)	<0.001	5.51 (4.57–6.71)	<0.001
IV	18.66 (6.13–46.25)	<0.001	11.66 (9.65–14.10)	<0.001

Abbreviations: CI, confidence interval; ESS, Endometrial stromal sarcoma; HR, hazard ratio; SCC, Shanghai Cancer Center; SEER, Surveillance, Epidemiology, and End Results; UPS, undifferentiated pleomorphic sarcoma.

Previous studies have reported that the survival rates of STS vary among different anatomic sites, and deep tumors are associated with worse outcomes than superficial tumors.^7^, ^19^, ^20^ In this study, STS originating from superficial organs (breast, testis, or penis) were excluded, and all STSs originated from deep organs. However, whether prognosis shows a difference according to different visceral organ systems remains unclear. Our database showed that there was no significant difference in survival between the different organ systems (*p* = 0.72, Figure S5A). However, the survival probability was significantly correlated with the organ system in the SEER database (*p* < 0.001, Figure S5B), and visceral sarcoma of the female genital system had a favorable prognosis, whereas visceral sarcoma of the respiratory system showed an adverse outcome. Similar prognoses were observed for visceral sarcomas of the digestive system, urinary system, and other systems (Figure S5B). Further analysis revealed that visceral sarcoma of the female genital system had the lowest proportion of high‐grade tumors and lymph node metastases, and the highest proportion of stage I. In contrast, visceral sarcoma of the respiratory system showed the highest proportion of high‐grade tumors, lymph node metastasis, distant metastasis, and stage IV disease, and the lowest proportion of stage I disease (Table S3). However, in our cohort, the proportions of high‐grade tumors, tumor size, distant metastasis, and stage II, III, and IV disease among the respiratory, urinary, digestive, and female genital systems were approximately evenly distributed (Table S4). In addition, the proportions of high‐grade tumors, tumor size, lymph node metastasis, distant metastasis, and stage I, II, III, and IV disease between the urinary and digestive systems were evenly distributed in the SEER cohort (Table S3). These results suggest that the prognosis of visceral sarcoma can be stratified by the TNM staging system, regardless of the site in which it is located.

### 3.4 Comparison of FIGO staging system with the visceral sarcoma staging system for female genital sarcoma

**FIGURE 5:**
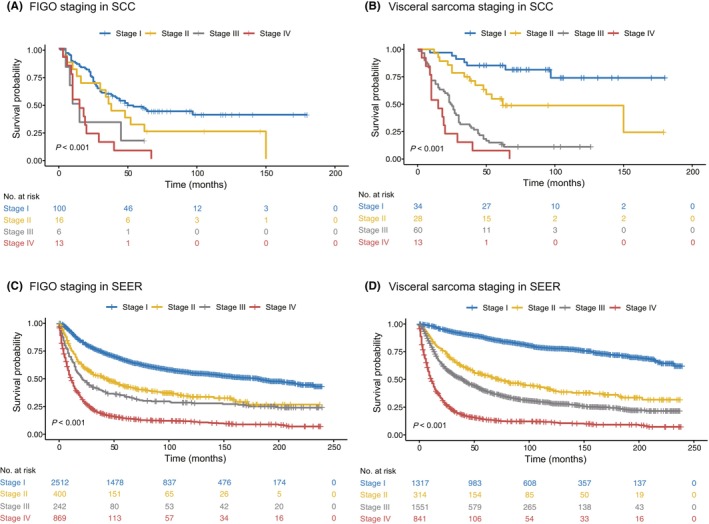
Comparison of the FIGO staging system with the visceral sarcoma staging system for female genital sarcoma. The patients with female genital sarcoma from the SCC and SEER databases were, respectively, classified by FIGO staging system (A, C). The same patients from the SCC and SEER databases were, respectively, re‐classified by the visceral sarcoma staging system (B, D).

Currently, there are few staging systems for visceral sarcoma, but visceral sarcoma of the female genital system is an exception. The FIGO staging system is currently widely used for female genital sarcoma.^12^, ^14^, ^21^ To clarify which staging system was more appropriate, we compared the FIGO staging system with the visceral sarcoma staging system (Table S2). In both cohorts, the prognoses of the four stages were not well stratified according to the FIGO staging system (Figure 5A,C). Stage I included a large proportion of patients (74.1% from SCC and 62.4% from SEER). Patients with stage II and III STS had similar long‐term survival in SEER (Figure 5C). However, the visceral sarcoma staging system (Table S2) dramatically differentiated patients into different prognostic groups (Figure 5B,D). For SCC, the 5‐year survival rate was 85.3% for stage I in the visceral sarcoma staging system and 46.2% for stage I in the FIGO staging system. Consistent with the results, in the validation SEER cohort, the 5‐year survival rate for stage I in the visceral sarcoma staging system was higher than that in the FIGO staging system (87.4% vs. 66.6%, respectively. Table S5). Furthermore, the visceral sarcoma staging system stratified patients more evenly across stages than the FIGO staging system in both cohorts. Moreover, the multivariate analysis showed that the visceral sarcoma staging system was superior to the FIGO staging system with regard to HR not only in the SCC series but also in the SEER series (Table S6). Taken together, these results show that the visceral sarcoma staging system is better than the current FIGO staging system for staging female genital sarcoma.

## 4 DISCUSSION

STS is a rare and heterogeneous disease,^19^ that occurs at various sites in the human body. STS arising from the viscera has worse outcomes than those arising from the extremity/trunk.^7^, ^22^ One of the important reasons for this is that there is no available staging system for visceral sarcoma. An accurate staging system can provide prognostic information, stratify patients by risk, and assist in therapeutic decision making. Thus, there is an urgent need to develop a staging system for visceral sarcomas. In this study, using our SCC cohort (*n* = 203) and the SEER validation cohort (*n* = 5826), we innovatively established a staging system (Table S2) to stratify the prognosis of visceral sarcoma and further confirmed that the visceral sarcoma staging system is superior to the currently available FIGO staging system for female genital sarcoma.

Tumor size is an important prognostic factor that participates in tumor staging in STS from the trunk/extremity or retroperitoneum.^23^, ^24^ However, the currently proposed NCCN T classification for STS of the abdomen and thoracic visceral organs stratifies the local tumor extent based on extra organ extension.^10^ Unfortunately, studies have noted that the T classification is problematic.^7^ In our cohort, T stages were not well separated by organ‐invasion T classification. Moreover, a large proportion of patients were classified as T1 in our cohort and SEER cohort (70% and 60.2%, respectively). Interestingly, while the T classification was based on tumor size, the patients were more evenly distributed among the subgroups, and the prognosis of T1 according to tumor‐size classification was superior to that of T1 based on organ‐invasion T classification (Figure 1). In accordance with our results, tumor size is an important prognostic indicator in patients with genitourinary sarcoma, pulmonary sarcoma, or uterine leiomyosarcoma.^3^, ^4^, ^21^ These results indicate that size‐based definitions are more objective.

In the SCC and SEER data series, visceral sarcoma arising from the female genital system accounted for a large proportion (66.5% and 70.9%, respectively). The FIGO staging system has been frequently applied to this disease.^17^, ^21^ However, the FIGO staging system cannot effectively and reliably stratify patients into four different prognostic groups,^13^, ^14^, ^25^ which was also shown in this study (Figure 5A,C). This may be because STS is a very different disease entity that behaves differently from epithelial carcinoma. Currently, the FIGO staging system for STS is similar to that for epithelial neoplasms from the uterus, emphasizing the importance of adjacent tissue invasion in prognosis.^26^ It is also not reasonable for sarcoma and epithelial tumors from the ovary or fallopian tube to both be classified by the same staging system.^27^ Tumor size and grade are important prognostic indicators of STS arising from the female genital system.^12^, ^13^, ^14^, ^28^ In this study, the visceral sarcoma staging system with parameters of tumor size, histologic grade, and lymph node metastasis stratified patients more evenly with different prognostic groups than the FIGO staging system in the SCC data and SEER validation data (Figure 5). In addition, stage I disease has a much better 5‐year survival rate than FIGO staging (85.3% vs. 46.2% in SCC; 87.4% vs. 66.6% in SEER). These results confirm that the visceral sarcoma staging classification (Table S2) is more applicable to female genital sarcoma than the current FIGO staging system.

We acknowledge several limitations in the current study. Firstly, this study was limited by its retrospective nature. Secondly, sarcomas from the chest wall or abdominal wall were excluded in this study. In addition, further studies with a large sample size are needed to validate the new STS staging system for visceral sarcoma (especially for female genital sarcoma).

In conclusion, tumor size, grade, and lymph node metastasis were important prognostic factors for sarcoma of the abdomen and thoracic visceral organs, which was confirmed not only in our cohort but also in the SEER validation cohort. Based on these prognostic factors, we established a new staging system for visceral sarcoma, by which patients could be stratified into clinically meaningful and non‐overlapping stages in both our cohort and the SEER validation series. In addition, the staging system is better than the current FIGO staging system for female genital sarcoma. The visceral sarcoma staging system can be considered for inclusion in future editions of the AJCC Cancer Staging Manual to stratify patients with STS of the abdomen and thoracic visceral organs.

## AUTHOR CONTRIBUTIONS

**Bingnan Wang:** Writing – original draft (lead). **Lun Xu:** Software (equal). **Meng Fang:** Data curation (lead). **Biqiang Zheng:** Writing – review and editing (lead). **Wangjun Yan:** Formal analysis (lead).

## FUNDING INFORMATION

This study was supported by grants from the National Natural Science Foundation of China. Grant Nos.81472480 (W.Y.) and 81302103(B.Z.).

## CONFLICT OF INTEREST STATEMENT

The authors declare no conflict of interest.

## Supporting information


Data S1.

## Data Availability

All data used and code developed for these analyses are available on Github at: https://github.com/HexStark/StickExcel.
