# Supplementary material for: Establishment of a staging system for visceral sarcoma
Source: Cancer Med. 2023 Dec 16;13(1):e6791. doi: 10.1002/cam4.6791 (PMC10807571; doi:10.1002/cam4.6791)
Supplement: Supplementary file 1 — Data S1. [file CAM4-13-e6791-s001.docx]

**Supplementary materials:**

**Detailed information of patient selection process of US Surveillance, Epidemiology, and End Results (SEER) 17 cancer registries:** The SEER database was used in this study. Patients with sarcomas of the abdomen and thoracic visceral organs from 2000 to 2017 were included in the study. Histological codes from the International Classification of Diseases for Oncology, 3rd edition (ICD-O-3), were used to identify patients with sarcoma. The correspondences between the codes and histological diagnoses were as follows: sarcoma (8800), spindle cell sarcoma (8801), giant cell sarcoma (8802), small cell sarcoma (8803), epithelioid sarcoma (8804), undifferentiated sarcoma (8805), fibrosarcoma (8810), fibromyxosarcoma (8811), myxosarcoma (8840), liposarcoma (8850), liposarcoma, well differentiated (8851), myxoid liposarcoma (8852), round cell liposarcoma (8853), pleomorphic liposarcoma (8854), mixed liposarcoma (8855), fibroblastic liposarcoma(8857), dedifferentiated liposarcoma (8858), leiomyosarcoma (8890), epithelioid leiomyosarcoma (8891), angiomyosarcoma (8894), myosarcoma (8895), myxoid leiomyosarcoma (8896), rhabdomyosarcoma (8900), pleomorphic rhabdomyosarcoma (8901), mixed type rhabdomyosarcoma (8902), embryonal rhabdomyosarcoma (8910), spindle cell rhabdomyosarcoma (8912), alveolar rhabdomyosarcoma (8920), rhabdomyosarcoma with ganglionic differentiation (8921), endometrial stromal sarcoma (8930), endometrial stromal sarcoma, low grade (8931), adenosarcoma (8933), embryonal sarcoma (8991), synovial sarcoma (9040), synovial sarcoma, spindle cell (9941), synovial sarcoma epithelioid cell, (9042), synovial sarcoma, biphasic (9043), clear cell sarcoma (9044), hemangiosarcoma (9120), Ewing sarcoma (9260). TNM information was retrieved based on the following codes: Combined Summary Stage 2004, SEER Combined Summary Stage 2000 (2004-2017), collaborative stage (CS), tumor size 2004, CS extension 2004, CS lymph nodes 2004, CS metastases at DX 2004, extent of disease (EOD) 10-extent (1988-2003), EOD 10-nodes (1988-2003), and EOD 10-size (1988-2003).

According to site recode ICD-0-3/WHO 2008, the anatomical sites that we selected in SEER database were as follows: digestive system (excluding retroperitoneum, peritoneum, omentum and mesentery), lung and bronchus, female genital system (excluding vagina and vulva), prostate glands, urinary system and adrenal gland.

Patients were excluded using the same criteria as those for our SCC cohort: (1) the sarcoma was an organ metastasis, not primary; (2) the tumor was diagnosed as a gastrointestinal stromal tumor or carcinosarcoma; or (3) the tumor originated from superficial organs (breast, testis, or penis). Based on the initial search results, 14280 patients were screened. The exclusion criteria are shown in Figure S1.


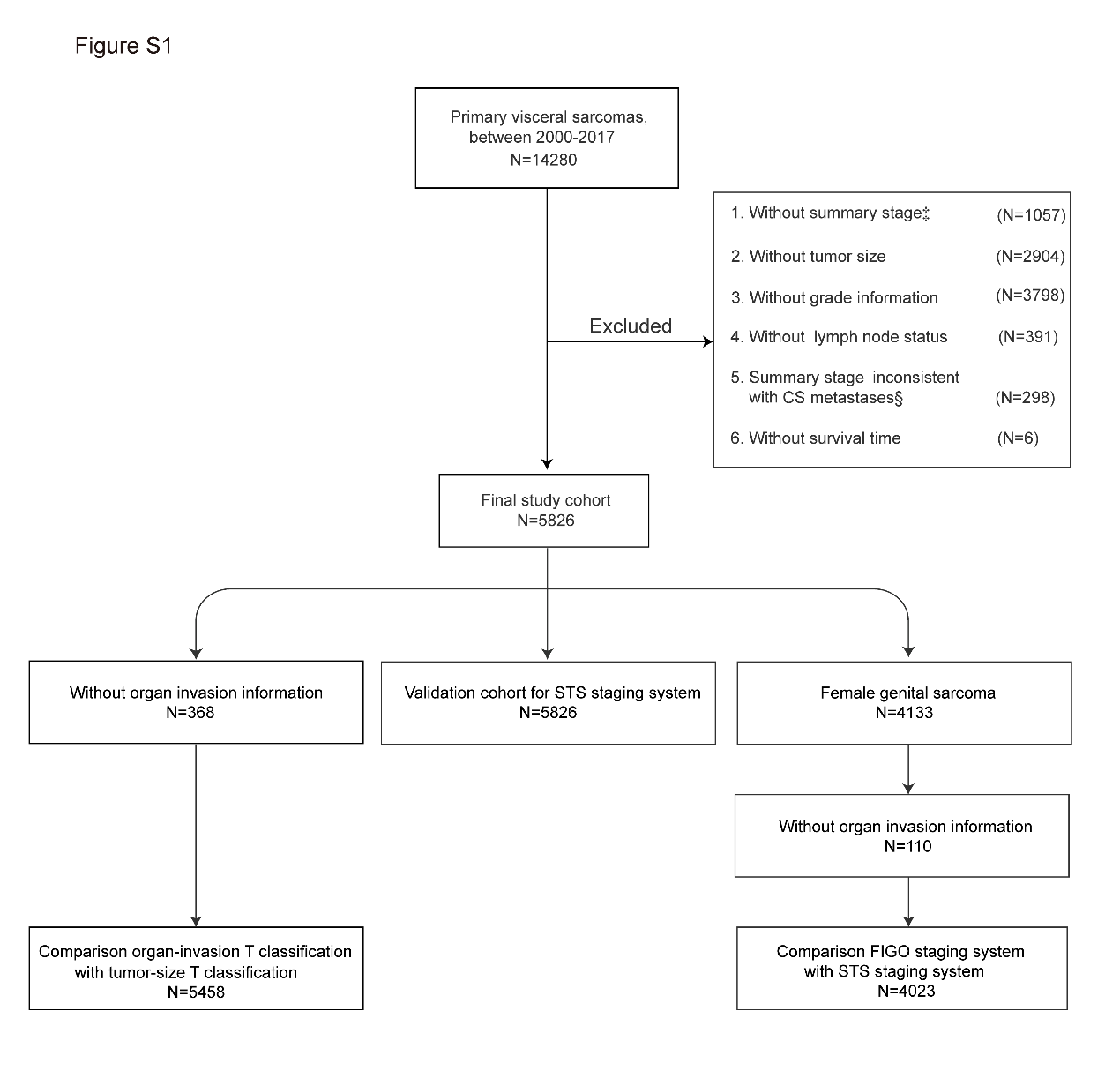


**Figure S1.** Flow chart of patient selection from the SEER database.

‡ Summary stage includes four types of information: localized, regional, distant, and blank; patients with blank information were excluded.

§ CS metastases: collaborative stage metastases.


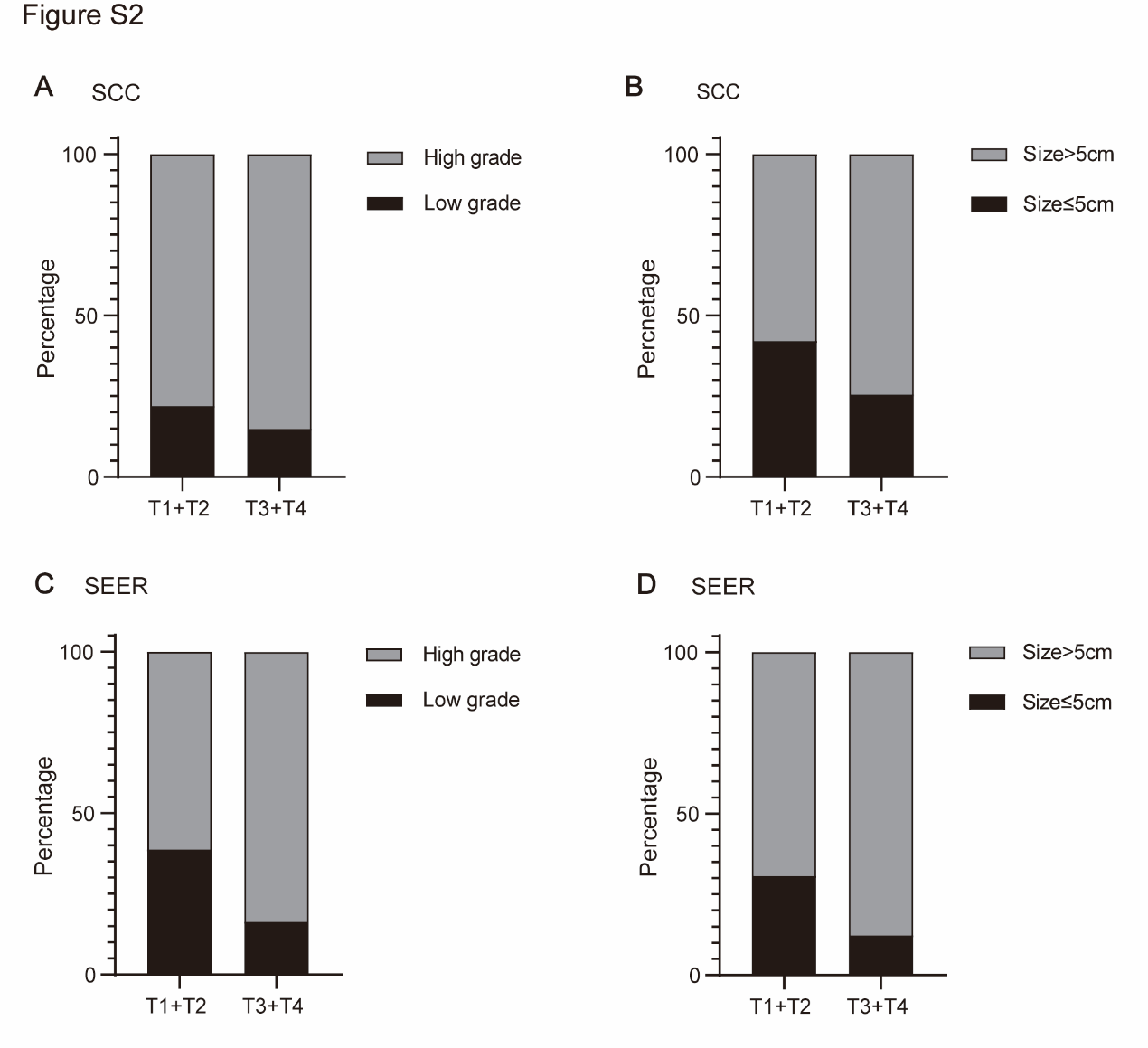


**Figure S2:** High T (T3+T4) was associated with large tumor size and high grade in visceral sarcomas stratified by organ-invasion T classification.


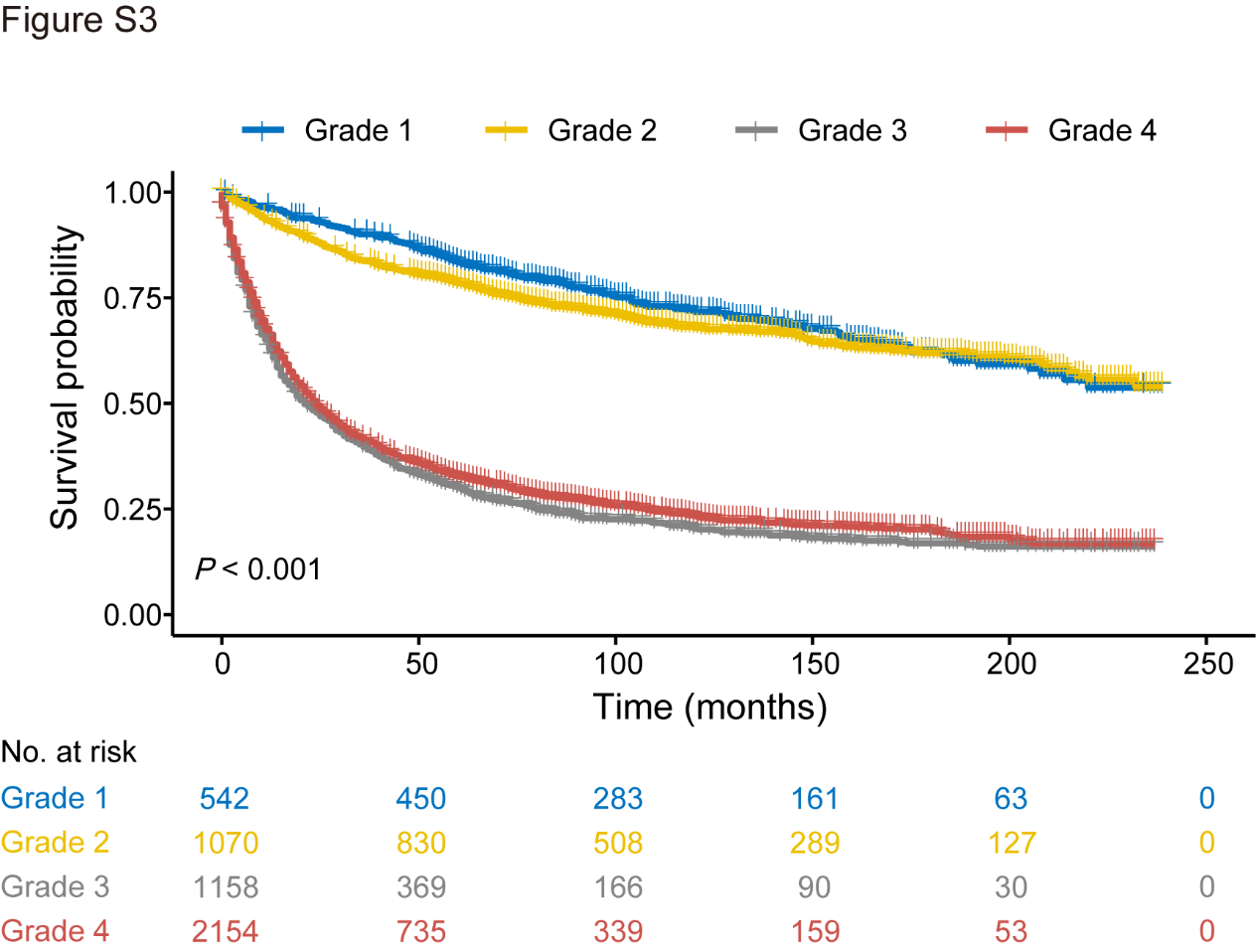


**Figure S3:** The prognosis of G1, G2, G3, and G4 patients in the SEER cohort.


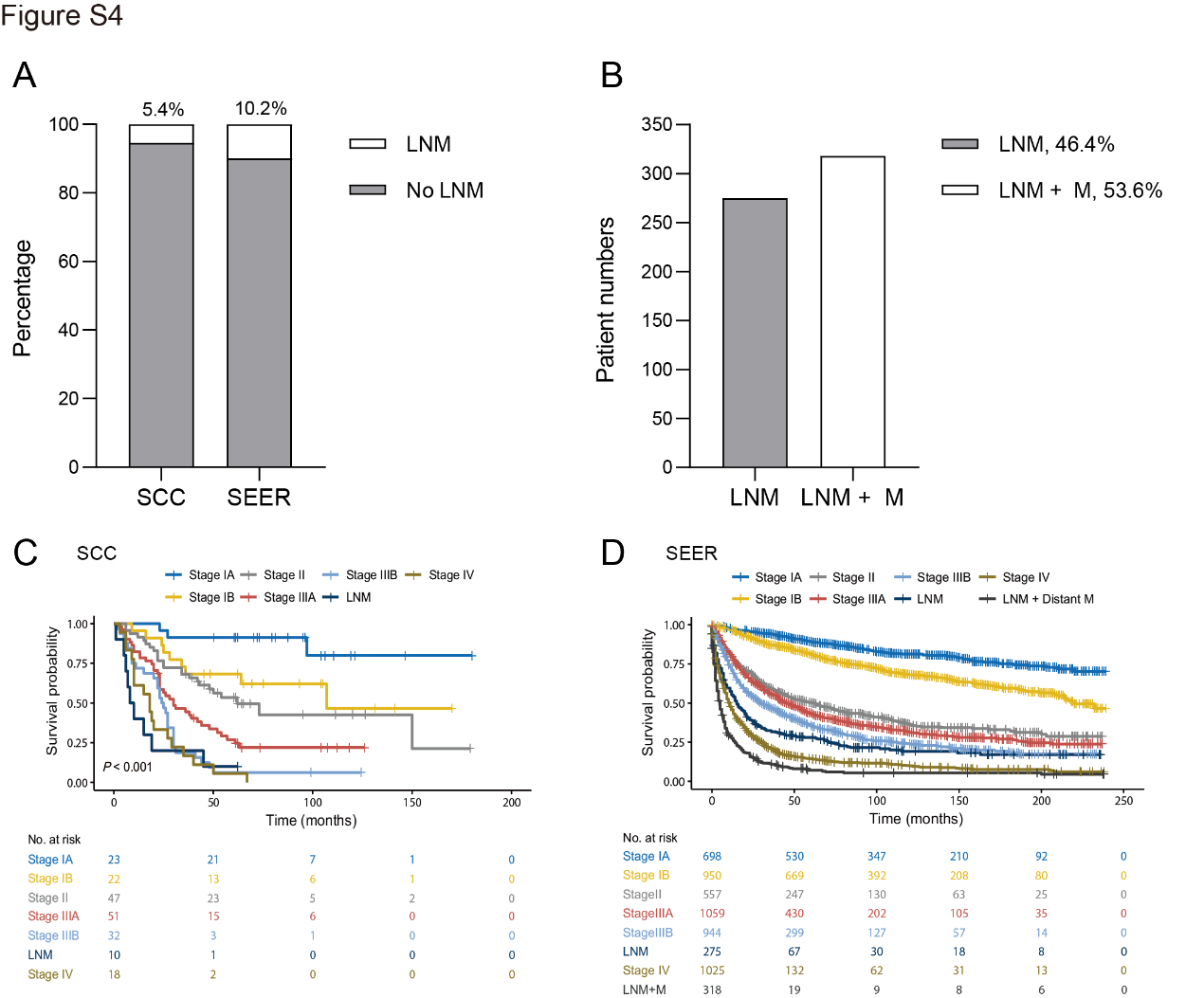


**Figure S4:** The analysis of lymph node metastasis (LNM) in the prognosis of visceral sarcoma. (A) The proportion of LNM in SCC and SEER. (B) The number of LNM and LNM + distant metastasis (M) in the SEER database. The prognosis of LNM in the SCC (C) and SEER cohorts (D).


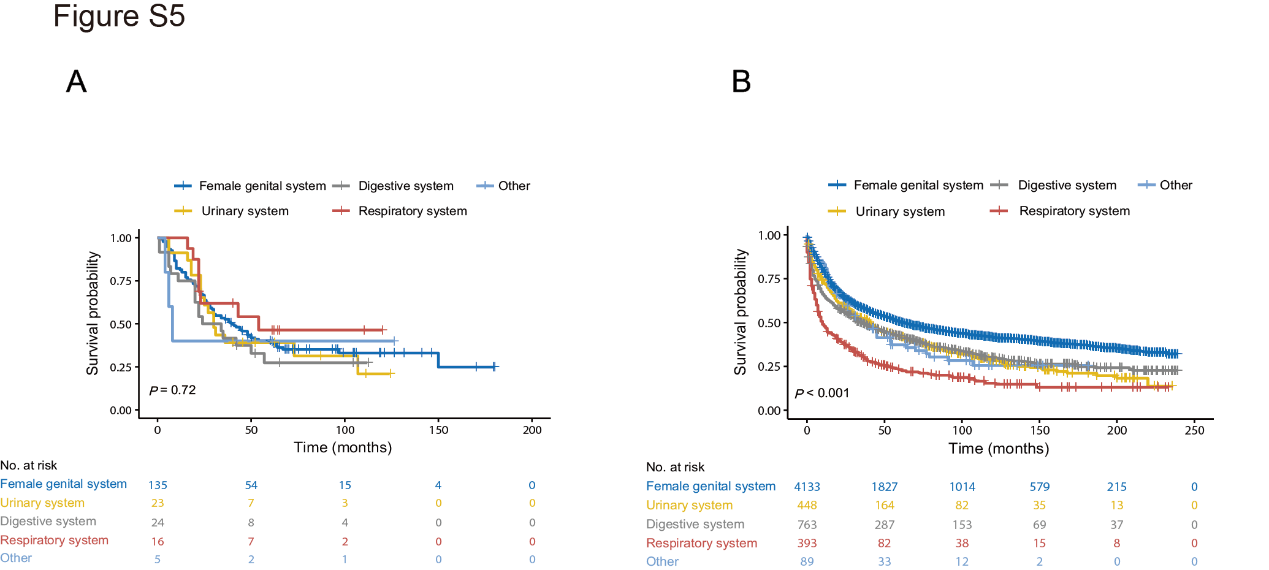


**Figure S5:** The prognosis of patients with visceral sarcoma according to different organ systems from the SCC (A) and SEER databases (B).

**Table S1:** Sarcomas from uncommon sites with histopathology

| Rare site | Histological type |
| --- | --- |
| Prostate | Leiomyosarcoma |
| Prostate | Ewing sarcoma |
| Adrenal gland | Leiomyosarcoma |
| Ureter | Leiomyosarcoma |
| Spleen | Hemangiosarcoma |
| Esophagus | UPS |
| Esophagus | Leiomyosarcoma |
| Appendix | UPS |

UPS: Undifferentiated pleomorphic sarcoma

**Table S2:** Establishment of a new staging system for visceral sarcoma (Visceral sarcoma staging system)

| Stage | Definition | | | |
| --- | --- | --- | --- | --- |
| TX | Primary tumor cannot be assessed | | | |
| T0 | No evidence of primary tumor | | | |
| T1 | Tumor 5 cm or less in greatest dimension | | | |
| T2 | Tumor more than 5 cm and less than or equal to 10 cm in greatest dimension | | | |
| T3 | Tumor more than 10 cm | | | |
| N0 | No regional lymph node metastasis or unknown lymph node status | | | |
| N1 | Regional lymph node metastases | | | |
| M0 | No distant metastasis | | | |
| M1 | Distant metastases | | | |
| Stage | T | N | M | G |
| Ⅰ A | 1 | 0 | 0 | G1, GX |
| Ⅰ B | 2 or 3 | 0 | 0 | G1, GX |
| Ⅱ | 1 | 0 | 0 | G2, G3 |
| Ⅲ A | 2 | 0 | 0 | G2, G3 |
| Ⅲ B | 3 | 0 | 0 | G2, G3 |
|  | Any T | 1 | 0 | Any G |
| Ⅳ | Any T | Any N | 1 | Any G |

**Table S3:** The characteristics of patients with visceral sarcoma according to different organ systems from the SEER database.

| Site | Patient number | High grade | Tumor  >5cm | LNM | Distant M | Stage I | Stage II | Stage III | Stage IV |
| --- | --- | --- | --- | --- | --- | --- | --- | --- | --- |
| Respiratory system | 393 | 327 (83.2%) | 239  (60.8%) | 117 (29.8%) | 155 (39.5%) | 41  (10.4%) | 65 (16.5%) | 132 (33.6%) | 155 (39.5%) |
| Urinary  system | 448 | 342  (76.3%) | 346  (77.2%) | 39  (8.7%) | 84  (18.8%) | 97 (21.6%) | 58 (12.9%) | 209 (46.7%) | 84 (18.8%) |
| Digestive system | 763 | 563 (73.8%) | 542  (71.0%) | 102  (13.4%) | 184 (24.1%) | 165 (21.6%) | 101 (13.3%) | 313 (41.0%) | 184 (24.1%) |
| Female genital  system | 4133 | 2653 (64.2%) | 3162 (76.5%) | 326 (7.9%) | 909 (22.0%) | 1326 (32.1%) | 318 (7.7%) | 1580  (38.2%) | 909 (22.0%) |
| Others | 89 | 69  (77.5%) | 64  (71.9%) | 9  (10.1%) | 11  (12.4%) | 19 (21.3%) | 15 (16.9%) | 44 (49.4%) | 11  (12.4%) |

**Table S4:** The characteristics of patients with visceral sarcoma according to different organ systems from the SCC database.

| Site | Patient number | High grade | Tumor  >5cm | LNM | Distant M | Stage I | Stage II | Stage III | Stage IV |
| --- | --- | --- | --- | --- | --- | --- | --- | --- | --- |
| Respiratory system | 16 | 15 (82.1%) | 10  (62.5%) | 1  (6.25%) | 1  (6.2%) | 1  (6.2%) | 5 (31.2%) | 9  (56.4%) | 1  (6.2%) |
| Urinary  system | 23 | 17 (73.9%) | 15  (65.2%) | 0 | 2  (8.7%) | 6 (26.1%) | 5 (21.7%) | 10 (43.5%) | 2  (8.7%) |
| Digestive system | 24 | 21 (87.5%) | 16  (66.7%) | 3  (12.5%) | 2  (8.3%) | 3 (12.5%) | 6 (25.0%) | 13 (54.2%) | 2  (8.3%) |
| Female genital system | 135 | 99 (73.3%) | 86 (63.7%) | 7  (5.2%) | 13  (9.6%) | 34 (25.2%) | 28 (20.7%) | 60 (44.5%) | 13 (9.6%) |
| Other | 5 | 4 (90.0%) | 1  (20%) | 0 | 0 | 1  (20%) | 3  (60%) | 1  (20%) | 0 |

**Table S5**. Comparison of the prognosis for female genital sarcoma by the FIGO staging system and STS staging system

|  | SCC SCC | | SEER SEER | |
| --- | --- | --- | --- | --- |
| Staging system | FIGO | STS | FIGO | STS |
| I | 46.2% | 85.3% | 66.6% | 87.4% |
| II | 25.0% | 48.8% | 42.8% | 53.3% |
| III | 16.7% | 13.3% | 33.5% | 39.6% |
| IV | 7.7% | 7.7% | 14.2% | 13.4% |

**Table S6:** Comparison of FIGO staging system with visceral sarcoma staging system for female genital sarcomas from SCC or SEER database by multivariate COX analysis

|  |  |  | **FIGO** **staging** |  | **Visceral sarcoma staging** | |
| --- | --- | --- | --- | --- | --- | --- |
| **Database** | **Variable** |  | **HR (95%CI)** | **P value** | **HR (95%CI)** | **P value** |
| **SCC** | Age | ≤50 | Reference |  | Reference |  |
|  |  | >50 | 1.92 (1.17-3.15) | 0.009 | 1.29 (0.80-2.10) | 0.16 |
|  | Histological type | Leiomyosarcoma | Reference |  |  |  |
|  |  | ESS | 0.22 (0.08-0.64) | 0.006 | 0.40 (0.28-1.42) | 0.10 |
|  |  | Adenosarcoma | 0.77 (0.41-1.43) | 0.40 | 1.00 (0.55-1.84) | 0.98 |
|  |  | Others | 1.79 (1.06-3.05) | 0.03 | 0.96 (0.56-1.65) | 0.89 |
|  | Stage | Ⅰ | Reference |  | Reference |  |
|  |  | Ⅱ | 1.71 (0.91-3.20) | 0.09 | 2.22 (1.20-7.46) | 0.02 |
|  |  | Ⅲ | 6.56(2.41-17.88) | <0.001 | 7.63 (3.93-18.03) | <0.001 |
|  |  | Ⅳ | 3.44 (1.79-6.60) | <0.001 | 15.02 (5.61-32.84) | <0.001 |
| **SEER** | Age | ≤50 | Reference |  | Reference |  |
|  |  | >50 | 1.96 (1.78-2.16) | <0.001 | 1.70(1.54-1.87) | <0.001 |
|  | Histological type | Leiomyosarcoma | Reference |  |  |  |
|  |  | ESS | 0.51 (0.46-0.56) | <0.001 | 0.74 (0.66-0.82) | <0.001 |
|  |  | Adenosarcoma | 0.64 (0.54-0.76) | <0.001 | 0.84 (0.71-0.99) | 0.05 |
|  |  | Others | 1.38 (1.23-1.57) | <0.001 | 1.41 (1.25-1.60) | <0.001 |
|  | Stage | Ⅰ | Reference |  | Reference |  |
|  |  | Ⅱ | 1.95 (1.70-2.30) | <0.001 | 2.80 (2.31-3.40) | <0.001 |
|  |  | Ⅲ | 2.54 (2.16-2.98) | <0.001 | 3.86 (3.35-4.46) | <0.001 |
|  |  | Ⅳ | 4.35 (3.94-4.80) | <0.001 | 9.66 (8.34-11.21 ) | <0.001 |

SCC: Shanghai Cancer Center; SEER: Surveillance, Epidemiology, and End Results; FIGO: International Federation of Gynecology and Obstetrics; HR: hazard ratio; CI: confidence interval; ESS: endometrial stromal sarcoma.
